# Charting the effects of TMS with fMRI: Modulation of cortical recruitment within the distributed network supporting semantic control

**DOI:** 10.1016/j.neuropsychologia.2016.09.012

**Published:** 2016-12

**Authors:** Glyn P. Hallam, Carin Whitney, Mark Hymers, Andre D. Gouws, Elizabeth Jefferies

**Affiliations:** Department of Psychology and York Neuroimaging Centre, University of York, YO10 5DD York, UK

**Keywords:** Semantic cognition, Inferior frontal gyrus, Posterior middle temporal gyrus, Transcranial magnetic stimulation, Executive function

## Abstract

Semantic memory comprises our knowledge of the meanings of words and objects but only some of this knowledge is relevant at any given time. Thus, semantic control processes are needed to focus retrieval on relevant information. Research on the neural basis of semantic control has strongly implicated left inferior frontal gyrus (LIFG) but recent work suggests that a wider network supports semantic control, including left posterior middle temporal gyrus (pMTG), right inferior frontal gyrus (RIFG) and pre-supplementary motor area (pre-SMA). In the current study, we used repetitive transcranial magnetic stimulation (1 Hz offline TMS) over LIFG, immediately followed by fMRI, to examine modulation of the semantic network. We compared the effect of stimulation on judgements about strongly-associated words (dog-bone) and weaker associations (dog-beach), since previous studies have found that dominant links can be recovered largely automatically with little engagement of LIFG, while more distant connections require greater control. Even though behavioural performance was maintained in response to TMS, LIFG stimulation increased the effect of semantic control demands in pMTG and pre-SMA, relative to stimulation of a control site (occipital pole). These changes were accompanied by reduced recruitment of both the stimulated region (LIFG) and its right hemisphere homologue (RIFG), particularly for strong associations with low control requirements. Thus repetitive TMS to LIFG modulated the contribution of distributed regions to semantic judgements in two distinct ways.

## Highlights

• Offline rTMS was used to modulate the semantic control system.
• fMRI revealed post-stimulation changes in other areas of the semantic control system.
• Semantic retrieval requires the flexible activation of representations shaped by control processes.

## 1 Introduction

Semantic cognition is central to our mental lives, allowing us to understand the meaning of words, objects, pictures and faces, and to use this knowledge to drive context- and time-appropriate behaviour (Corbett et al., 2009, Lambon Ralph and Patterson, 2008). As our concepts are embedded in a rich web of associations, only some of which will be relevant in a given task or context, semantic cognition involves at least two interacting components – a *store of conceptual knowledge*, plus *control mechanisms* that shape semantic processing to suit the context or task. For example, if we see a banana skin on the floor, we need to retrieve knowledge that this object is slippery and disregard irrelevant information about its sweet flavour (Jefferies, 2013, Jefferies and Lambon Ralph, 2006). Executive control over knowledge activation is vital for successful semantic cognition, yet the neural basis of this function is not well understood. In particular, functional neuroimaging studies have focused almost exclusively on the contribution of left inferior frontal gyrus (LIFG; Thompson-Schill et al., 1997; Badre and Wagner, 2007), while neuropsychological investigations (Jefferies and Lambon Ralph, 2006, Noonan et al., 2010, Corbett et al., 2009), neuroimaging meta-analyses (Noonan et al., 2013), and studies using inhibitory transcranial magnetic stimulation (TMS, Whitney et al., 2011, Whitney et al., 2012 and Davey et al., 2015), point to the possibility of a large-scale distributed network underpinning semantic control.

Comparisons of patients with multi-modal semantic deficits in the context of semantic dementia (SD) and semantic aphasia following stroke (SA) show that semantic representations and control processes can be selectively impaired. Central semantic representations are thought to be degraded in SD, producing loss of conceptual knowledge across the full range of modalities, e.g., vision, hearing, touch, and action (Patterson et al., 2007, Bozeat et al., 2000, Hodges et al., 2000). In contrast, SA is associated with deficient semantic control, resulting in poor comprehension across modalities despite a broadly intact knowledge base (Corbett et al., 2009, Jefferies and Lambon Ralph, 2006, Noonan et al., 2010, Novick et al., 2009, Thompson-Schill et al., 1998). SA patients with multimodal semantic deficits have large and variable lesions, typically affecting left prefrontal cortex (particularly ventral left inferior frontal gyrus; LIFG) and/or left temporoparietal regions, particularly posterior middle temporal gyrus (pMTG). Moreover, SA patients with prefrontal and temporoparietal infarcts show largely parallel deficits on tasks requiring high degrees of semantic control: they have difficulty establishing semantic relationships between weakly associated words, avoiding strong distracters, understanding the non-dominant meanings of ambiguous words (Noonan et al., 2010) and identifying non-canonical uses of objects (e.g., understanding that a newspaper can be used to swat a fly; Corbett et al., 2009).

These findings are consistent with the view that semantic control is underpinned by a *large-scale distributed cortical network* including both left ventral prefrontal and posterior regions although SA patients typically have large lesions making it difficult to precisely localise the critical regions for this deficit. Converging evidence is provided by fMRI studies of healthy participants, which often reveal activation within similar distributed brain regions when semantic control demands are manipulated (Badre et al., 2005, Noonan et al., 2013, Thompson-Schill et al., 1997, Wagner et al., 2001). For example, a recent meta-analysis based on activation likelihood estimation (ALE) revealed that brain activity in left and right IFG, left pMTG, pre-SMA and dorsal angular gyrus (dAG) bordering the intraparietal sulcus (IPS) was reliably associated with high control demands across a range of different semantic tasks (Noonan et al., 2013). This network is distinct from, yet partially overlapping with, the multiple-demand network which underpins executive control (Duncan, 2010): ventral LIFG and pMTG appear to have a relatively selective semantic focus, while dorsal PFC and IPS contribute to domain-general executive control (Davey et al., 2016, Krieger-Redwood and Jefferies, 2014, Noonan et al., 2013, Whitney et al., 2012). Regions implicated in semantic but not domain-general control may play a particularly crucial role in controlled memory retrieval: i.e., situations in which there is no explicit goal specifying which aspects of meaning must be selected, yet automatic spreading activation between related concepts is insufficient for efficient task performance. Under these circumstances, it is the activation of conceptual representations that gives rise to the control demands (Davey et al., 2016, Jefferies, 2013). An example might be retrieving weak associations: the dominant aspects of meaning are likely to be irrelevant for identifying the context that links weakly-related words together and so control must be employed to focus retrieval on information relevant to this linking context. Research suggests that these controlled retrieval mechanisms also support the retrieval of weak episodic memories (Barredo et al., 2015).

Studies examining the effect of inhibitory TMS to LIFG and pMTG in healthy participants provide causal evidence for a role of these regions in controlled semantic retrieval (Hoffman et al., 2010, Davey et al., 2015, Whitney et al., 2011, Whitney et al., 2012). When TMS pulses are applied repeatedly at a low frequency, the effects last beyond the end of the stimulation period: in this ‘offline’ method, effects of TMS are assessed *following* rather than during stimulation, suggesting that behavioural disruption reflects changes to cortical recruitment as opposed to distraction caused by scalp sensations, eye-blinks and jaw contractions. We previously found that offline TMS to LIFG and pMTG produced comparable disruption of tasks tapping semantic control (Whitney et al., 2011). There were no TMS effects on judgements about strong associations (with low control demands) at either of these sites.

While there is increasingly strong evidence that semantic control is supported by a distributed network that includes regions beyond LIFG, such as pMTG and pre-SMA, little is currently known about the way in which damage or disruption to one brain region (e.g., LIFG) modulates the contribution of another site to semantic control (e.g., pMTG; pre-SMA). TMS, when combined with neuroimaging techniques, can be used to investigate effective connectivity and modulation within large-scale neural networks (Paus, 2005, Ruff et al., 2009, Zanto et al., 2011, Bestmann et al., 2004, Sack et al., 2007). This type of modulation effect could be critical to understanding both TMS effects in healthy participants and the effects of brain lesions in neuropsychological cases. Stimulation of LIFG might *reduce* activity within connected brain regions; alternatively, if pMTG and LIFG form a single flexible functional network, there might be *increases* in pMTG which could help task performance to be maintained at a good level despite stimulation of LIFG.

In the present study, we used a combination of TMS and fMRI to establish *how* a distributed network of brain regions is recruited in a flexible manner to support semantic control. Offline TMS was applied to ventral LIFG (or, in a separate testing session, a control site at the occipital pole) and fMRI was used to measure the subsequent effect of this stimulation on brain activity in regions implicated in semantic control by a recent meta-analysis (Noonan et al., 2013). This was done both for weak associations requiring controlled retrieval (which might reveal increases in recruitment across a distributed network following the application of TMS to LIFG) and strong associations with lower controlled-retrieval demands (which should be possible without an efficient contribution of LIFG). By comparing cortical activity and functional connectivity following perturbation of the LIFG with a perturbation of a control site, for both strong and weak associations, we examined modulation of the network that underpins semantic control (cf. Paus, 2005; Ruff et al., 2009).

## 2 Materials and methods

### 2.1 Participants

Imaging and behavioural data from 18 right-handed, native English speakers was examined (13 female; M age=22.5 years, SD=3.2). All participants were students from the University of York and passed TMS and MRI safety screening (Wassermann, 1998). Written informed consent was obtained from each subject before testing and a reimbursement of £30 was paid. The study was approved by the local ethics committee.

### 2.2 Experimental procedure and task

Participants were scanned three times, with the sessions separated by at least one week. In the first session, the anatomical scan was acquired plus functional images from the relatedness judgement task (baseline scan). In the second and third sessions, participants performed the same tasks again during fMRI but received 15 min of TMS to either LIFG or occipital pole (OP; control site) before undergoing scanning. The LIFG stimulation site was defined for each participant by identifying a local peak response in this region in the baseline scan, while the OP stimulation site was defined using structural landmarks (see below for details). The order of stimulation sites was counterbalanced across subjects.

**Fig. 1:**
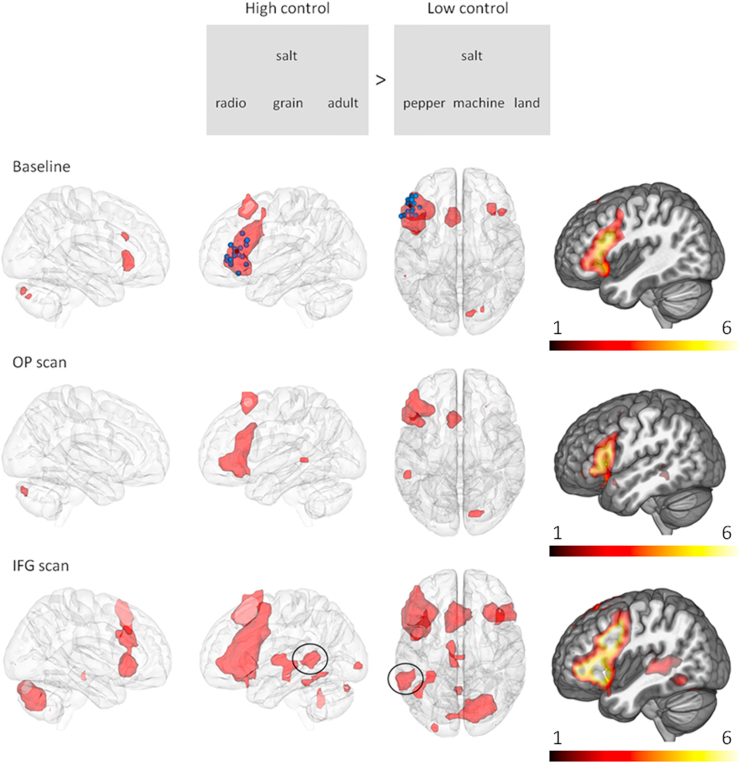
Brain activation for high controlled retrieval>low controlled retrieval during the baseline scan and after TMS was applied to OP (control site) and LIFG (experimental site), shown on a glass brain and also a rendered view (right-hand panel; colour bar represents t-values). Activation is corrected for multiple comparisons at p<.05, with a voxel type I error of p <.005. Blue dots represent the site for LIFG stimulation for each subject (group mean in black), which were based on individual brain activation during the high control condition in the baseline scan. Increased activity after TMS to LIFG in left posterior middle temporal gyrus (pMTG) is circled. Images were constructed using Data Viewer 3D (Gouws et al., 2009), and MRICroGL.

Two semantic judgement tasks with different levels of semantic control demand were employed: weak associations with high controlled retrieval demands, and strong associations with low controlled retrieval demands (Fig. 1) (cf. Badre et al., 2005; Wagner et al., 2001). In each task, a cue word appeared above a row of three potential target words. Participants were asked to decide which target was related to the cue by pressing one of three buttons with their left hand, corresponding to the position of the response item (left, middle, right). When the target was strongly related to the cue (e.g. salt – pepper, machine, land), automatic spreading activation between the probe and target is thought to support the matching process. In contrast, when cue-target associations were weaker (e.g. salt – grain, radio, adult), retrieval may need to be controlled in order to focus on those aspects of the cue and probe words that are relevant to the link between them. In these trials, it was more difficult to select the target and reject the distracters..

### 2.3 Stimuli

A within-subject factorial design was used, with FMRI SESSION (baseline, OP and IFG scan) and SEMANTIC CONTROL (strong and weak associations) as within-subject factors. Stimuli were selected for each of the two relatedness tasks from a previous investigation (Whitney et al., 2011) and split into sets of 50 items per condition. The strong and weak association trials were constructed such that the same cue word was matched with a closely or more distantly related semantic associate, using several sets of association norms (Moss and Older, 1996, Postman and Keppel, 1970). Association strength was defined as the proportion of subjects that named the target in response to the cue in free association. Each cue word was also paired with two unrelated distracter items, for which no entry in the association norms was found (e.g., low control: salt – pepper, machine, land; high control: salt – grain, radio, adult). The mean association strength for high and low control cue-target pairs differed significantly (paired *t*-test: low=.24, SD=.19; high=.03, SD=.04; *t*(149)=13.34; *p*<.001), whereas cue, target and distracter items were matched for word length in letters and frequency (Kucera and Francis, 1967) across conditions (paired *t*-tests, *t*<1.34). The same cue was never repeated within a set/session and the assignment of stimulus set was counterbalanced across sessions.

### 2.4 fMRI procedure

In each of the three fMRI sessions, strong and weak associations were presented in mini-blocks, alternating with 7 s of rest (i.e., fixation). We constructed 10 blocks for each experimental condition, containing 5 trials each, and 21 blocks of rest. Each experimental block started with an alertness cue (‘!’) shown for 1 s, which was replaced by a fixation cross shown for 500 ms in the centre of the screen, which was followed by the first trial displaying the cue and its three response options. As soon as the participant pressed a button to denote which target was related to the cue (relatedness judgement task), the fixation cross reappeared for 500 ms indicating the next trial. If no response was detected within 5 s, the fixation cross appeared automatically. The task was self-paced and participants took on average 6:38 min (SD=29 s), 6:24 min (SD=24 s) and 6:30 min (SD=29 s) to complete the tasks during the baseline, the OP and the IFG scan, respectively.

Presentation of stimuli was controlled by a computer using the Presentation 10.1 software package (Neurobehavioral Systems, http://www.neurobs.com/). Stimuli were back-projected onto a screen located inside the magnetic bore, viewable through a mirror mounted above the head coil. Responses were recorded using an MRI-compatible button-box.

### 2.5 Data acquisition

For each subject, T2*-weighted axial EPI scans, parallel to the AC/PC line, were acquired with a GE 3 T HD Excite MRI scanner using a Magnex gradient insert head coil together with a birdcage, radio-frequency coil. 160 functional volumes were recorded in each session (number of slices=39; slice thickness=3.5 mm; matrix size=128×128; field of view=288×288 mm; TE=32.5 ms; TR=3 s). In addition, a T1-weighted anatomical image (1 mm×1 mm×1 mm) was acquired for each subject, which was used to guide coil positioning during TMS (see below).

### 2.6 fMRI data analysis

Pre-processing and statistical analyses were performed using Statistical Parametric Mapping software (SPM8) implemented in MATLAB (Mathworks Inc., Sherborn, MA). After discarding the initial two volumes, images were realigned to the first image and unwarped to correct for the interaction of movement and susceptibility artifacts during image acquisition. Each slice was then shifted relative to the acquisition time of the middle slice using a sinc-interpolation. Volumes were normalised into standard stereotaxic anatomical MNI-space by using the transformation matrix calculated from the first EPI-scan of each subject and the EPI-template. Afterwards, the normalised data with a resliced voxel size of 4×4×4 mm were smoothed with an 8 mm FWHM isotropic Gaussian kernel to accommodate intersubject variation in brain anatomy. The time series data was high-pass filtered with a high-pass cut-off of 1/128 Hz. The autocorrelation of the data was estimated and corrected for.

For each subject, the pre-processed images from all three sessions (the baseline, OP and IFG scan) were entered as separate sessions into the same design matrix. For each session, the strong and weak association conditions were modelled as box-car functions with variable duration, starting from the presentation of the first trial in each sequence to the beginning of the resting block. Each of these functions was then convolved with the expected hemodynamic response, defined as the canonical hemodynamic response function (HRF) (Friston et al., 1998) and its temporal derivative, to create covariates in a general linear model. Parameter estimates of the HRF regressors for each of the six different conditions were calculated from the least mean squares fit of the model to the time series.

A random-effects analysis was performed on the group data by entering the six 1st level contrasts for each subject into a factorial analysis of variance (ANOVA) with factors FMRI SESSION (baseline, OP and IFG scan) and SEMANTIC CONTROL (strong and weak associations). We were interested in how activation might change after TMS to LIFG within the distributed neural network supporting semantic control; hence between-session contrasts were computed on the task with high semantic control demands involving weakly-associated words (i.e., IFG scan vs. Baseline scan, IFG scan vs. OP scan). To ensure that any observed effects could be attributed to regions involved in semantic control processes, we computed the same contrasts for the judgements about strongly-associated words with low control demands (i.e., IFG scan vs. Baseline scan, IFG scan vs. OP scan). Results for the whole brain analysis are presented at a threshold of p<.05 FWE corrected.

We also conducted a further analysis in which we added a parametric regressor (Büchel et al., 1998) to model the effects of time since TMS stimulation. Each task block at the individual level was given a demeaned parametric regressor (number of seconds since stimulation). The resulting images were then analysed in a 2×2 model looking at the IFG and OP scans only (since time since stimulation only applied to these sessions). This analysis allowed us to look at which brain areas changed in activation as a function of time since stimulation, over and above any existing task effect.

Since we had clear predictions about which cortical areas beyond LIFG contribute to semantic control from the meta-analysis of Noonan et al. (2013), we supplemented our whole-brain analysis with a regions-of-interest (ROI) analysis. We examined neural responses to high and low-control judgements in the five sites that were the most likely to be recruited across a wide variety of semantic control manipulations in this meta-analysis: these sites were left IFG, left pMTG, pre-SMA, dorsal AG bordering IPS and right IFG, listed in order of activation likelihood according to Noonan et al. (2013). We selected ROIs individually for each participant within these pre-defined anatomical areas using the contrast of high>low control in the *baseline* scan (in the absence of TMS). We placed 10 mm spheres around peak activations for individual participants, ensuring that these peaks were within the anatomical region of interest as defined by the automatic anatomical labelling (AAL) templates. We then examined the response of these sites in the LIFG and OP scans (therefore the data used to define the ROI and the percent signal change values extracted from the ROI were independent). ROIs were successfully created for individual participants using this method in left pMTG, left and right IFG, and pre-SMA. However, it did not prove possible to reliably identify activation for high>low-control judgements within the dorsal portion of AG bordering IPS for individual participants: there was typically little signal or deactivation to this contrast. Although dAG/IPS was implicated in semantic control by the Noonan et al. (2013) meta-analysis, it has been suggested that its contribution is more restricted to tasks involving selection or requiring the application of a top-down goal to retrieval and that it does not strongly respond to manipulations of associative strength (Badre et al., 2005). Therefore this region was not included in the ROI analysis below. For the other four sites, data were extracted using the MarsBar toolbox in SPM8 (Brett et al., 2002) and effects of stimulation site (LIFG vs. OP stimulation) and semantic control (high vs. low-control judgements) were examined using within-subjects ANOVA.

#### 2.6.1 Connectivity analysis

In order to further investigate the effects of stimulation on the large-scale networks supporting semantic control, we conducted a psychophysical interaction (PPI) analysis (O’Reilly et al., 2012), in which we investigated differences in connectivity between the stimulation sessions (following TMS to LIFG and the control site), and conditions (strong vs. weak associations). We extracted the time-course (for each participant and each session) from 5 mm spheres centred on the LIFG stimulation site (individually defined for each participant). We compared the functional connectivity of LIFG with a control site in medial prefrontal cortex (mPFC), in order to test whether the effects of task and/or stimulation on connectivity were relatively *specific* to LIFG, or whether they would generalise to other nearby regions outside the semantic control network. This specific control site was chosen since it fell within prefrontal cortex yet shows anti-correlation with LIFG in functional connectivity analyses (see Supplementary Fig. 1). The mPFC coordinates were taken from Andrews-Hanna et al. (2010), who identified this region as corresponding to a peak within the default mode network. Consequently, mPFC represents a region that is not functionally coupled to LIFG, falls outside the network identified as important for semantic control by the neuroimaging meta-analysis of Noonan et al. (2013), and might be expected to show a higher response to easy as opposed to hard semantic judgements (Davey et al., in press). In the supplementary materials, we also present parallel PPI analyses employing the occipital pole as a control site (see Supplementary Fig. 2).

In both of these models, eigenvariates for these sites were included in a GLM model as explanatory variables at the single-subject level, and brain regions whose activity was associated with the time-course for these spheres were identified. These were combined at the second-level across participants in the same fashion as the whole-brain analysis of the BOLD response to the task. Results were thresholded at p<.05 FWE corrected.

### 2.7 TMS protocol

In the second and third fMRI sessions, TMS was applied over either OP or LIFG before participants underwent scanning. We employed an offline ‘virtual lesion’ rTMS protocol, which was compatible with established TMS safety guidelines (Rossi et al., 2009, Wassermann, 1998). Repetitive trains of TMS (rTMS) were delivered at 1 Hz to the target brain area for 15 min. This type of repetitive stimulation is reported to produce a temporary disruption of neural processing in the underlying tissue, lasting for around the same length of time as the stimulation – i.e., 15 min (Lambon Ralph et al., 2009, Pascual-Leone et al., 1998, Pobric et al., 2007, Whitney et al., 2011). Stimulation intensity was determined before each rTMS administration as 100% of active motor threshold (MT). MT was identified as the lowest intensity that produced a visible muscle twitch in the tense right hand when intensity was gradually decreased during single-pulse stimulation of left motor cortex. Intensity threshold was set to a maximum of 60% of stimulator output (mean intensity OP scan =55%, SD =6.30; mean intensity IFG scan =55%, SD =5.78). We previously employed more intense stimulation (delivered at 120% not 100% of active MT) for a shorter duration (10 not 15 min) to disrupt behavioural performance employing the same tasks (Whitney et al., 2011). However, our current stimulation parameters were optimised for detecting modulation of the neural response in fMRI (as opposed to behavioural disruption) since we needed to ensure that the effects of stimulation would be present throughout the functional scan: for this reason we opted to stimulate for a longer period, at a reduced intensity to maintain the comfort and safety of participants.

A 50 mm figure-of-eight coil, attached to a Magstim Rapid2 stimulator, was used for the repetitive delivery of magnetic pulses. The centre of the coil was aligned to the point that marked the stimulation site on a tight-fitting elastic cap worn by the participant. The coil was supported by a portable coil stand and held firmly against the scalp throughout stimulation. TMS was administered in the MRI control room to minimise the time delay between stimulation offset and acquisition of the first functional image (mean time delay OP scan: 3:21 min; SD =24 s; range: 2:48–4:20 min; mean time delay IFG scan: 3:28 min; SD =21 s; range: 2:49–4:00 min). Therefore, the period of functional data acquisition (which corresponded approximately to the period 3–11 min after TMS stimulation ended) was expected to fall within the period of TMS-induced cortical modulation (Table 1).

### 2.8 Localization of stimulation sites

The stimulation site for OP was defined using structural landmarks, as lying 2 cm above the inion. The stimulation site for LIFG was determined for each participant individually based on their brain activation during the baseline scan and their structural image. For each subject, MNI-coordinates for LIFG were extracted from the 1st level contrast high control > rest, as this condition placed the highest demands on the semantic control network. Activation peaks were chosen such that they lay within BA 45 of the pars triangularis (according to the Anatomy toolbox labelling) or, if no peak emerged in this area, more ventrally within the pars orbitalis. The peak with the highest Z-value was chosen. The mean coordinates correspond to x =−49, x =30, z =9 (SD: x =4.3 mm, y =6.76 mm, z =10.34 mm) and were located in left BA 45 of the pars triangularis (see Fig. 1). A ‘Brainsight’ frameless stereotaxy system was used to co-register the identified site within LIFG to the participant's head. Each individual anatomical image was overlaid on the MNI template and the subject-specific stimulation site was marked. In a second step, the participant's head was co-registered with the anatomical image using a Polaris infra-red tracking device and five standard landmarks (i.e., nasion, tip and bridge of the nose, left and right ear). The target areas were marked on a tight-fitting elastic cap worn by the participant throughout stimulation.

## 3 Results

### 3.1 fMRI analysis: whole-brain analysis

Judgements of strong and weak associations at baseline, after OP stimulation, and after stimulation of LIFG, resulted in brain activations in highly similar, distributed, bilateral regions (see Table 2). Compared to rest, brain activity was consistently seen across all six conditions (i.e., strong and weak associations, within baseline, LIFG and OP TMS scans) in visual cortex, adjacent occipito-temporal cortex, left fusiform gyrus and left pMTG. Activity spread along the superior parietal lobe to sensory-motor areas and into LIFG. Right frontal responses occurred consistently in the insula, the middle frontal gyrus and, corresponding to left-hand button-presses, in large portions of right motor cortex and surrounding areas.

Contrasts of weak>strong associations were used to identify regions that respond to controlled semantic retrieval. During the baseline scan, differential activation occurred in areas previously associated with semantic control, including LIFG, left pMTG, left and right supplementary motor area, ventral right inferior frontal gyrus (RIFG) and the right cerebellum (see Table 3, Fig. 1). After OP stimulation – which should not have altered activation in the semantic control network – activation was seen in the same set of areas apart from the right supplementary motor area. After TMS to LIFG, responses to the high > low control contrast increased in the same set of areas (although to different degrees and in several different ways, revealed by the ROI analysis below). Additional activation was observed in left fusiform gyrus, bilateral inferior occipital gyrus and midbrain structures in the LIFG TMS scan. However, in the whole brain analysis, the interaction between session and control demands did not reveal any significant clusters.

An additional analysis that included a parametric regressor of time since stimulation (comparing LIFG and OP sessions, and excluding the baseline scan when no stimulation was applied) confirmed these findings. There was still no interaction between session and controlled retrieval demands, and no effect of time since stimulation for either site.

### 3.2 ROI analysis

**Fig. 2:**
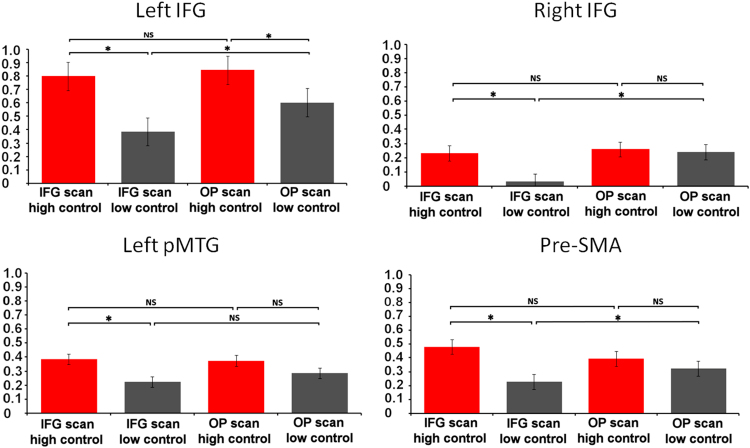
Results of the Region of Interest (ROI) analyses.

Signal change for the high and low control conditions during the IFG and OP scans was extracted for each participant within a sphere centred on the contrast high > low control in the baseline scan. This data was analysed using 2×2 ANOVA, examining within-subjects factors of semantic control demands and scan session. These data are shown in Fig. 2, along with a summary of the results of Bonferroni-corrected pair-wise comparisons computed between the four conditions at each site..

#### 3.2.1 Left inferior frontal ROI

LIFG (mean MNI co-ordinates=−51, 25, 10) showed a main effect of control (F(1,17)=48.124, p <.001) and a main effect of session (F(1,17)=4.642, p <.05). There was also a trend-level interaction between control and session (F(1,17)=3.533, p=.08). Paired *t*-tests (with Bonferroni correction adjusting p to <.0125) showed that neural activity in LIFG was substantially greater for high control compared to low control judgements in both sessions (IFG: t =5.323, p=.0001; OP: t=4.759, p=.0001). There was a reduced response to strong association trials with low control demands following stimulation of LIFG compared to OP (t=−3.714, p=.002), which might have reflected the local inhibitory effect of the stimulation. There was no difference in percentage signal change for weak association judgements with higher control demands following LIFG and OP stimulation (t <1).

#### 3.2.2 Left middle temporal ROI

Left pMTG (mean MNI co-ordinates =−56,−47, 2) showed a main effect of control (F(1,17)=14.667, p =.002). There was also an interaction between session and control demands (F(1,17)=4.636, p =.048). Paired *t*-tests (with Bonferroni correction adjusting p to <.0125) confirmed that neural activity in pMTG was higher for high control compared to low control judgements following IFG stimulation (t =3.905, p =.001) with a near-significant effect of control demands in the scan following OP stimulation (t =1.897, p =.077). This pattern of results is consistent with the possibility that activity in pMTG made a greater contribution to demanding semantic judgements following perturbation of the LIFG.

#### 3.2.3 Right inferior frontal ROI

RIFG (mean MNI co-ordinates=48, 26, 9) showed a main effect of control (F(1,17)=9.383, p =.008) and a main effect of session (F(1,17)=6.482, p <.022). There was also a significant interaction between control and session (F(1,17)=5.509, p=.033). Bonferroni-corrected *t*-tests showed a stronger response in RIFG for high compared to low control judgements only in the IFG session (t=3.394, p=.004) and not following OP stimulation (t <1). There was a reduced response in RIFG for low control judgments following stimulation of LIFG compared with stimulation of OP (t =−3.041, p=.008). However, for high control judgements, there was no difference between LIFG and OP stimulation (t <1). Thus, TMS to LIFG reduced the contribution of this region to relatively easy tasks, but this effect was not seen for harder judgements.

#### 3.2.4 Pre-supplementary motor area ROI

The pre-SMA (mean MNI co-ordinates =−6, 19, 57) showed a main effect of control (F(1,17)=7.712, p =.014) and an interaction between session and control (F(1,17)=6.79, p =.02). Paired *t*-tests showed that activity was stronger during high control compared to low control judgements only in the IFG session (t =3.513, p =.003) and not the OP session (t =1.104, p =.287). However, differences between the sessions did not reach significance for either high-control (t =1.106, p =.286) or low-control trials (t=−1.441, p=.170).

#### 3.2.5 Connectivity analysis

**Fig. 3:**
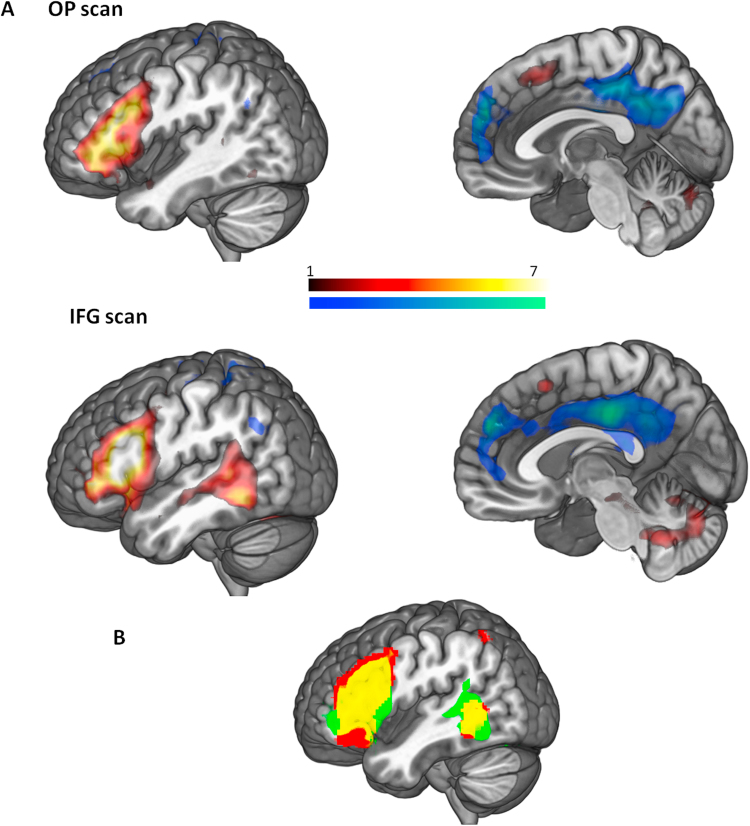
A. Brain activation for PPI analysis comparing connectivity of the IFG stimulation site with the mPFC control seed (red/yellow) and the inverse contrast (blue/green). Colour bars represent t values. **B.** Overlap of IFG connectivity in IFG scan with binorised mask of the Noonan meta-analysis of high>low semantic control, showing overlap in the networks.

We investigated functional connectivity during the task with psychophysical interactions that examined the whole-brain connectivity of the LIFG stimulation site, for weak and strong associations. We compared this effect at the stimulation site to a control region selected to be relatively close to the stimulation site but in a different functional network (mPFC in the default mode network), as this allowed us to demonstrate the spatial selectivity of the results. Across all three sessions, LIFG showed greater connectivity to surrounding voxels in LIFG and inferior frontal sulcus, left pMTG, RIFG, and pre-SMA compared to the mPFC seed (Fig. 3), during the task compared to the implicit baseline. The reverse contrast revealed greater connectivity from mPFC to other areas in the default-mode network (posterior cingulate, bilateral angular gyri). There were no main effects or interactions involving task condition (high > low control demands) or session (LIFG > OP stimulation). Taken together, these results suggest that performance on the semantic association task is supported by the distributed semantic control network, including all of our ROIs taken from Noonan et al.’s (2013) meta-analysis. While components of this network appear to change the strength of their recruitment in response to LIFG stimulation (i.e., in the analyses of the BOLD response above), we did not observe evidence that the network itself significantly changes. Indeed, LIFG shows a similar pattern of functional connectivity to sites implicated in semantic control in resting-state data (Davey et al., in press; see also Supplementary Fig. 1)..

### 3.3 Behavioural analysis

We analysed median response time (RT) to reduce the influence of outlying values (Wilcox and Keselman, 2003). We examined error rate and RT with incorrect trials and outliers (±2 SD) removed (9.63% of the data was discarded for this reason). The data were entered into repeated-measures ANOVAs with FMRI SESSION (Baseline, OP and IFG scan) and SEMANTIC CONTROL (strong vs. weak associations) as within-subject factors. 2-tailed paired *t*-tests were used for post-hoc analyses.

The ANOVA for median RT revealed a main effect of SEMANTIC CONTROL (F(1, 17)=114.864, p <.001), with longer RTs for the task with high as opposed to low semantic control demands. Individual comparisons confirmed that participants were slower for the high control than the low control task during the baseline scan (high control: M =1603 ms, SD =288; low control: M =1373 ms, SD =208; t(17)=7.08, p <.001), the OP TMS scan (high control: M =1530 ms, SD =250; low control: M =1316 ms, SD =162; t(17)=6.74, p <.001) and the IFG scan (high control: M =1562 ms, SD =283; low control: M =1355 ms, SD =222; t(17)=8.25, p <.001). There was a significant effect of FMRI SESSION (F(1,17)=5.015, p <.05, Baseline session: M =1488 ms, SD =244; OP session: M =1423 ms, SD =211; IFG session M =1458 ms, SD =260). Individual comparisons showed that median reaction times were slower in the baseline scan compared to the OP scan (p<.05) with no difference between the baseline and IFG scan (p=.522) or between the OP scan and IFG scan (p=.286) There was no interaction between SEMANTIC CONTROL and FMRI SESSION (F <1). Participants were slowest on the baseline session and this is likely to have reflected the fact that this session was always the first time they attempted the task.

We also investigated whether the reaction time changed as a result of time since the stimulation (see page 19). To investigate this, we split each session into the first half and last half. We then entered these into a separate ANOVA for each session with SEMANTIC CONTROL and TIME (1st half or 2nd half) as within subject factors. For the baseline scan there was a main effect of semantic control (F(1,17)=70.28, p<.001), with reaction times faster for low control than high control. There was no main effect of time (1st half or 2nd half; F(1,17)=1.508, p=.237) and no interaction between control and time (F(1,17)=.828, p=.376). For the OP scan there was a main effect of semantic control (F(1,17)=73.89, p<.001), with reaction times faster for low control than high control. There was no main effect of time (1st half or 2nd half; F(1,17)=.014, p=.907) and no interaction between control and time (F(1,17)=.603, p=.449). For the IFG scan there was a main effect of semantic control (F(1,17)=74.22, p<.001), with reaction times faster for low control than high control. There was no main effect of time (1st half or 2nd half; F(1,17)=.184, p=.184). However, there was an interaction between control and time (F(1,17)=6.862, p=.019): low control judgments were faster in the second half of the scan compared to the first (t=5.092, p<.001), but there was no difference for high control judgments in the first half and second half (t =−.388, p=.703). An additional omnibus ANOVA that included the effects of semantic control, time, and scan as within-subject factors revealed no interaction between semantic control, time and scan session (*F*(1,17)=.981, *p*=.39).

Although participants made relatively few errors, ANOVA examining accuracy revealed the same main effect of semantic control (*F*(1, 17)=48.19, *p*<.001). Participants were less accurate for high control than the low control trials during the baseline scan (high control: M =7.22%, SD =5.58; low control: M =3%, SD =3.16; *t*(17)=3.22, *p*=.005), the OP scan (high control: M =6.44%, SD =4.83; low control: M =3.89%, SD =4.01; *t*(17)=3.00, *p*=.008) and the IFG scan (high control: M =5.78%, SD =4.80; low control: M =3.11%, SD =3.01; *t*(17)=3.17, *p*=.006). No other main effects or interactions were significant (*F*<1).

## 4 Discussion

TMS-induced modulation of cortical activity can be observed even in the absence of behavioural disruption, and this method has been used to elucidate neurophysiological relationships between distant brain regions (Paus, 2005, Ruff et al., 2009, Zanto et al., 2011, Bestmann et al., 2004, Sack et al., 2007). We applied TMS to a key site for semantic control (LIFG) and measured the impact on neural recruitment using fMRI. We compared brain activity following stimulation of LIFG and a control site (occipital pole), confirming that modulation of the BOLD signal was site-specific. We found task-dependent modulation of the BOLD response in right IFG, posterior middle temporal gyrus (pMTG) and pre-SMA; regions which all show greater activation when healthy individuals make semantic judgement with high as opposed to low controlled retrieval demands in the absence of TMS (Badre et al., 2005, Thompson-Schill et al., 1997, Wagner et al., 2001, Noonan et al., 2013). These regions showed two distinct patterns of modulation following stimulation of LIFG: (i) effects of the semantic control manipulation (strength of association) were magnified in left pMTG and pre-SMA; (ii) the response of LIFG and RIFG was reduced in magnitude, particularly for the easy, strong-association condition. In the discussion that follows, the contribution of each of these sites to semantic control is discussed.

### 4.1 LIFG

In the whole brain fMRI analysis, off-line stimulation of LIFG did not produce any significant local effects (cf. Chouinard et al., 2003; O’Shea et al., 2007; Ruff et al., 2008), presumably reflecting the fact that we applied TMS to a functional peak that was anatomically unique for each participant. Since participant-specific LIFG stimulation sites were not spatially aligned, local changes in activation induced by TMS might have been spread out across the whole region. The region of interest analysis took this variation into account by identifying individual activation peaks (in the baseline scan without TMS, using the high > low control contrast). An ROI centred around these peak coordinates showed reduced signal change to semantic judgements in the context of LIFG stimulation, relative to OP stimulation (i.e., overall TMS had a local inhibitory effect on the BOLD signal). Moreover, neural recruitment of LIFG showed a trend-level interaction between control demands and stimulation site: the response in this region following stimulation to LIFG might have been better maintained for weaker associations that required greater control.

The global reduction in the BOLD response following TMS was expected, given that we used an inhibitory stimulation protocol that was expected to reduce recruitment of the underlying brain area (e.g. Binney and Lambon Ralph, 2015). However, our observation of greater TMS-induced changes for low-control items at LIFG was unexpected. We did observe stronger recruitment of LIFG for harder trials, in line with the literature, and a reduced response following stimulation, but this effect of stimulation did not interact with difficulty in the manner that we predicted. It is unclear why the recruitment of LIFG showed the biggest reduction for easy trials. There are still relatively few studies combining neuroimaging with offline TMS protocols (e.g. Binney and Lambon Ralph, 2015; Paus, 2005; Ruff et al., 2009; Zanto et al., 2011; Bestmann et al., 2004; Sack et al., 2007) and further work is needed to establish if this pattern will emerge across sites and tasks. While we cannot provide a complete interpretation of this pattern of results, we were able to show that response times to the low control trials decreased over time in the LIFG stimulation session, presumably because retrieval became more automatic when participants were more experienced at the task. High-control trials that required the retrieval of weak associations were unable to benefit from task practice in the same way. If TMS applied to LIFG reduced the efficiency of controlled retrieval processes as expected, participants may have been encouraged to adopt a more automatic retrieval strategy for the low-control trials. When a similar TMS protocol was applied to LIFG (albeit at a higher intensity) in an earlier study (Whitney et al., 2011), there was behavioural disruption of weak but not strong associations, suggesting that LIFG may not be *essential* for the efficient retrieval of strong associations, which may be retrieved via automatic spreading activation between highly-related concepts. If LIFG does not make an essential contribution to low-control trials, its engagement might be more readily reduced following inhibitory TMS. However, differences in the TMS protocol prevent us from directly comparing these studies and further research is clearly needed to replicate and investigate the pattern we observed.

We also examined the pattern of functional connectivity for LIFG using psychophysical interaction models. LIFG and pMTG showed an increase in their coupling during the semantic task, supporting the view that these regions act together to support semantic cognition; however, we did not observe changes in the structure of this network following IFG stimulation or for high-control vs. low-control trials. It might be that this analysis lacked the sensitivity to uncover such effects. Alternatively, TMS might have produced quantitative changes in the recruitment of nodes within this network *without* significantly altering the structure of the network itself: even when the BOLD response in LIFG was reduced post-stimulation, fluctuations in this response could still be correlated with fluctuations in the signal in pMTG. Recent research has shown that functional connectivity between executive and default mode regions can increase during a control-demanding semantic task, even when these regions show opposite patterns in BOLD (i.e., an increased BOLD response in PFC and deactivation in the default mode; Krieger-Redwood et al., 2016). Consequently, if participants adopted a more ‘automatic’ strategy for easy semantic trials following inhibitory TMS to LIFG, the neural basis of this effect may have been reduced signal in the stimulated region without a change in the correlation with pMTG.

### 4.2 RIFG

Although control-demanding semantic decisions elicit activity in a largely left-lateralised network, there is also significant recruitment of right IFG when judgements requiring more control are contrasted with more automatic semantic retrieval (Noonan et al., 2013), and thus we included this region as an ROI. RIFG showed a significant interaction between semantic control demands and site of stimulation (LIFG vs. OP), which again reflected *reduced* recruitment for easier judgements following LIFG stimulation. This finding can be considered within opposing theoretical frameworks about the contribution of left and right IFG to semantic processing (see Geranmayeh et al., 2014). By one view, RIFG is independently recruited alongside LIFG for more demanding judgements when additional semantic control is required. However, this proposal is not consistent with our data, since it fails to explain why RIFG activation was reduced following LIFG stimulation. Other accounts suggest that the balance of activity within LIFG and RIFG reflects inter-hemispheric interactions (e.g., Seghier et al., 2011, Chiarello and Maxfield, 1996), which could be inhibitory or might reflect the transfer of information (Bloom and Hynd, 2005). Our data are not readily explained by the principle of interhemispheric inhibition since a reduction in activation in LIFG for the easy task following TMS to this region elicited the same pattern in RIFG. Instead, our findings are more consistent with the proposal that LIFG and RIFG show *coupled* activity – thus TMS-induced modulation of LIFG would be expected to elicit similar effects in these two regions. Consistent with this pattern, studies have shown that executively demanding tasks which recruit PFC such as working memory and voluntary emotion regulation, may benefit from bilateral processing (see Geranmayeh et al., 2014; Buhle et al., 2013; Jansma et al., 2004; Niendam et al., 2012).

### 4.3 pMTG

In the neuroimaging meta-analysis of Noonan et al. (2013), left pMTG showed highly reliable recruitment across tasks that tapped semantic control in different ways – second only to LIFG. Therefore, LIFG and pMTG are recruited together when semantic retrieval must be steered away from dominant and automatically retrieved aspects of knowledge, towards more unusual features or associations (see also Davey et al., in press). LIFG and pMTG are highly interconnected: strong fibre pathways – running either ventrally via the extreme capsule/uncinate fasciculus (EC/UF) or dorsally via the arcuate fasciculus (AF) – allow the transmission of semantic information from posterior temporal to inferior frontal areas (Anwander et al., 2007, Croxson et al., 2005, Rilling et al., 2008, Saur et al., 2010).

However, the contribution of pMTG to semantic control remains controversial, largely because this site has alternatively been described as a key repository of semantic knowledge (Damasio et al., 1996, Martin, 2007, Small et al., 1995). In conventional fMRI studies, the greater neural response seen in pMTG during high-control semantic conditions might conceivably reflect additional activation of conceptual knowledge on demanding trials, as opposed to neural processing essential for semantic control; indeed, many researchers have adopted this interpretation (Badre et al., 2005, Bedny et al., 2008; see also Gennari et al., 2007; Gold and Buckner, 2002). Our previous research employing the same tasks has already shown that TMS to LIFG and pMTG can produce equivalent behavioural disruption for high-control but not low-control semantic decisions, strengthening the view that pMTG is necessary for efficient semantic control alongside LIFG (Whitney et al., 2011). However, semantic tasks are not process-pure (in that they always require stored representations to interact with control processes). The observation that TMS to LIFG magnified the effect of strength of association in the BOLD response in pMTG therefore provides critical support for the view that these regions are key sites within a flexible distributed neural system underpinning semantic control.

Our PPI results are also broadly compatible with this proposal, since they show that pMTG was coupled with LIFG during the semantic task, although we did not observe a modulation of this relationship with stimulation or semantic control demands. As noted above, the percentage signal change increases that were observed within pMTG for high control semantic judgements following TMS to LIFG, combined with an absence of stimulation effects in the PPI analysis, are consistent with the possibility that TMS produced quantitative changes in recruitment across the semantic network, but not changes to the structure of the network itself. However, these null results of task demands and stimulation might also have reflected our relatively short functional scan which was designed to fit within the period in which TMS effects were expected. Snijders et al. (2010) observed increased coupling between pMTG and large portions of bilateral anterior temporal lobes, left inferior and middle temporal gyri and fusiform gyrus when participants read semantically demanding ambiguous vs. unambiguous sentences. Strong correlations were found between left pMTG and ventral parts of LIFG (amongst other frontal areas), reflecting their common engagement in semantic control processes. Given pMTG's close proximity to temporal areas that store semantic representations/feature knowledge (e.g. Martin, 2007), yet strong connectivity with LIFG, this region might serve a complex role during semantic processing, mediating between storage and control regions and maintaining information about currently-relevant semantic features (Davey et al., in press).

### 4.4 Pre-SMA

The pre-SMA is involved in effortful cognitive control (Aron et al., 2007, Fedorenko et al., 2013, Harding et al., 2015) and is a component of the “multiple-demand” network (Duncan, 2010), supporting executively-demanding non-semantic tasks. Pre-SMA is also strongly engaged during semantic judgements requiring control over conceptual retrieval (see the meta-analysis of Noonan et al., 2013). The recruitment of this site in the current study was modulated by the application of TMS to LIFG in a similar way to pMTG: it showed a stronger response to the strength of association manipulation following LIFG stimulation, suggesting that this domain-general executive region may have been making a greater contribution to semantic control after an inhibitory TMS protocol was applied to a key semantic control site (LIFG).

### 4.5 dAG/IPS

There was no response to the task within dAG/IPS in the whole-brain analysis, even though this region is a putative part of the semantic control network and involved in broader cognitive control beyond the semantic domain (Dumontheil et al., 2011, Duncan, 2006, Nagel et al., 2008). Since ROIs were defined per participant using the contrast of high over low control in the baseline scan, and this contrast elicited little activation or deactivation at this site, we did not include dAG/IPS as an ROI. One possible explanation for this null result is that dAG is not critical for the type of controlled semantic retrieval required in the paradigm we used – instead, it might have a more specific role in the orientation of selective attention towards specific semantic features like shape, colour or size (Badre et al., 2005). Feature selection was not a major requirement of our high-control judgements, since the probe-target pairs were globally (though weakly) semantically related. In line with this interpretation, a previous TMS study showed that stimulation of dAG disrupted performance on a semantic feature selection task but not the weak association task used here (Whitney et al., 2012). Thus, different aspects of semantic control might recruit partially overlapping yet distinct neural networks.

### 4.6 Limitations

We acknowledge that the behavioural results did not reproduce the previously reported pattern of selective disruption of high control semantic judgments (Whitney et al., 2011); rather we found a main effect of TMS disruption for both types of semantic judgement that approached significance. There are several possible explanations for this weaker, non-significant effect. First, we applied stimulation at a lower intensity than in the previous study, and also for a shorter duration than some other studies that have used a combined TMS-fMRI approach (e.g. Rounis et al., 2006; Ward et al., 2010). Secondly, the novelty of the scanner environment may have resulted in both easy and harder judgements recruiting executive-semantic regions. Third, practical constraints, such as the reduced number of trials that we included in order to fit the task into a brief fMRI session within the period of TMS-induced disruption, may have reduced the sensitivity of our behavioural measure to subtle disruption. In any case, other studies have also reported modulation of neural activity following TMS in the absence of behavioural effects (e.g., O’Shea et al., 2007; Feredoes et al., 2011; Blankenburg et al., 2010; Bestmann et al., 2008).

In addition, although the OP provided a useful control site in that it was outside the semantic control network and thus not expected to modulate behaviour differentially according to the task demands, it was not equivalent to LIFG in terms of the perceived unpleasantness of stimulation and peripheral effects such as muscle twitches and eye blinks. Given that TMS was applied offline, these peripheral effects were not expected to directly influence task performance. Nevertheless, future research should examine double-dissociations between proximal brain regions that lie within different functional networks and are therefore expected to modulate the brain in distinct ways. A second point is that the OP stimulation site was determined using anatomical landmarks, as opposed to fMRI peak activation as for LIFG. Consequently, this site was less variable across participants and this could conceivably influence the effect of OP stimulation on the brain. We are unable to directly characterise the effects of OP stimulation, since we only employed one control site. However, our key analysis examines ROIs within the semantic control network which do not show strong connectivity to OP (Davey et al., in press). In contrast, the LIFG stimulation site selected for every participant was within the semantic control network of Noonan et al. (2013).

We also acknowledge that this study focussed on a specific aspect of semantic control – controlled semantic retrieval, i.e., the ability to identify relatively weak connections between probe and targets words that would not be accessed through relatively automatic patterns of spreading activation; it is possible that other aspects of semantic control, such as the selection of conceptual information relevant to a pre-encoded goal, is would not show the same pattern. For example, there is some evidence suggesting pMTG may be less important when specific semantic information has to be selected to suit a well-specified goal provided by the task instructions, as opposed to when semantic relationships defined by the input are weak or ambiguous (Davey et al., in press).

In sum, this study combines fMRI and TMS to provide evidence for a distributed semantic control network that extends beyond left prefrontal cortex. We show changes in the BOLD signal in several regions of the spatially-distributed semantic control network following offline stimulation to LIFG, including pMTG and pre-SMA: these sites are thought to contribute to semantic control and domain-general executive control respectively. We conclude that efficient semantic retrieval requires the flexible activation of semantic representations shaped by control processes to suit current task demands (Noonan et al., 2013) and perturbation of one component of the semantic control system (e.g., LIFG) results directly in changes within functionally connected components (e.g., pMTG).

## Figures and Tables

**Table 1:** Median RTs and standard deviations for the behavioural task.

					Median RT (ms)			SD	
**Baseline**			*Whole scan*						
		High control			1603			288	
		Low control			1373			208	
			*1*st *half of scan*						
		High control			1577			302	
		Low control			1371			163	
			*2*nd *half of scan*						
		High control			1650			294	
		Low control			1386			289	
**OP**			*Whole scan*						
		High control			1530			250	
		Low control			1316			162	
			*1*st *half of scan*						
		High control			1561			258	
		Low control			1302			146	
			*2*nd *half of scan*						
		High control			1540			277	
		Low control			1314			213	
**IFG**			*Whole scan*						
		High control			1562			283	
		Low control			1355			222	
			*1*st *half of scan*						
		High control			1558			289	
		Low control			1406			226	
			*2*nd *half of scan*						
		High control			1576			303	
		Low control			1308			218	
									

**Table 2:** Brain activation for the high control and low control conditions during the baseline scan and after TMS was applied to OP (control site) and LIFG.

**Activation peak**		**x**	**y**	**z**	***Z***	**Voxel**
**Baseline scan: high control**						
	Cerebellar vermis	4	−76	−28	>8	6498

*L*	*MOG (BA 17)*	*−16*	*−100*	*0*	*>8*	
*R*	*IOG (BA 17)*	*24*	*−100*	*−4*	*7.30*	
L	MTG (BA 21/22)	−60	−40	4	5.55	
L	SPL (BA 7)	−24	−68	48	7.60	
R	SPL (BA 7)	24	−60	56	5.67	
L	Precentral gyrus (BA 9)	−52	8	36	>8	
R	Precentral gyrus (BA 6)	40	−16	68	7.72	
L	IFG (tri; BA 44)	−44	16	24	7.47	
R	Insula	36	20	4	5.53	
L	Hippocampus	−28	−28	−4	5.53	44
R	MFG (BA 46)	56	28	32	4.13	25
L	Postcentral gyrus (IPC)	−56	−20	24	3.90	22
R	Precentral gyrus (BA 6)	60	8	36	3.75	9
R	Rolandic operculum	44	0	16	2.89	2

**Baseline scan: low control**						
	Cerebellar vermis	4	−76	−28	>8	7796
L	Lingual gyrus (BA 17)	−8	−80	−12	>8	
R	Lingual gyrus (BA 17)	8	−76	−12	>8	
L	MTG (BA 21/22)	−60	−44	8	5.27	
L	SPL (BA 7)	−24	−68	48	>8	
R	SPL (BA 7)	24	−60	56	6.65	
L	Precentral gyrus (BA 9)	−52	4	40	7.81	
R	Precentral gyrus (BA 6)	40	−16	68	>8	
L	IFG (op)	−52	12	0	5.47	
R	Insula	44	16	−4	4.56	
R	MFG (BA 46)	52	32	32	3.94	26
L	MTG (BA 21)	−60	−16	−4	3.27	5

**OP scan: high control**						
	Cerebellar vermis	4	−76	−28	>8	5412
L	MOG (BA 18)	−20	−100	0	>8	
R	Cuneus (BA 17)	0	−92	16	>8	
L	MTG (BA 22)	−60	−44	4	6.23	
L	SPL (BA 7)	−24	−64	44	>8	
R	SPL (BA 7)	24	−60	56	7.14	
L	Precentral gyrus (BA 9)	−52	8	36	>8	
R	Postcentral gyrus (BA 3)	40	−28	52	7.75	
L	IFG (tri; BA 44)	−44	16	24	>8	
R	Insula	36	24	4	6.61	
L	Postcentral gyrus	−56	−20	24	3.08	11
L	SMG	−48	−44	24	3.46	8
L	Midbrain	−12	−24	−20	3.08	3
R	Midbrain	8	−24	−20	2.83	3

**OP scan: low control**						
	Cerebellar vermis	4	−76	−28	>8	10409
L	Cuneus (BA 18)	0	−80	12	>8	
R	Cuneus (BA 17)	8	−88	4	>8	
L	MTG (BA 22)	−64	−32	0	4.98	
L	IPL	−24	−68	44	>8	
R	SPL (BA 7)	24	−60	56	>8	
L	Precentral gyrus (BA 9)	−52	4	36	>8	
R	Postcentral gyrus (BA 2)	44	−28	52	>8	
L	IFGtri	−40	16	24	7.73	
R	Postcentral gyrus	52	−20	20	5.33	

**IFG scan: high control**						
	Cerebellar vermis	4	−76	−24	>8	8050
L	Cuneus (BA 18)	0	−80	12	>8	
R	Lingual gyrus (BA 17)	12	−80	0	>8	
L	MTG (BA 21/22)	−60	−40	4	6.45	
L	SPL (BA 7)	−24	−64	44	>8	
R	SPL (BA 7)	24	−60	56	5.72	
L	Precentral gyrus (BA 9)	−48	8	36	>8	
R	Postcentral gyrus (BA 3)	44	−24	52	7.70	
L	IFGtri (BA 45)	−52	28	12	7.16	
R	MFG (BA 46)	56	28	32	5.96	
R	Insula	36	24	−4	5.61	
R	MTG (BA 21)	48	−32	0	3.60	16
R	IFG (tri)	36	32	28	2.74	2

**IFG scan: low control**						
	Cerebellar vermis	4	−76	−20	>8	7156
L	Calcarine gyrus (BA 18)	0	−80	12	>8	
R	Calcarine gyrus (BA 17)	28	−60	4	6.77	
L	MTG (BA 21/22)	−60	−40	4	5.21	
L	SPL (BA 7)	−24	−64	44	>8	
R	SPL (BA 7)	24	−60	56	5.98	
L	Precentral gyrus (BA 6)	−40	−4	64	>8	
R	Postcentral gyrus (BA 3)	44	−24	52	>8	
L	IFG (tri)	−40	20	24	5.57	
R	Insula	48	16	−4	3.72	
L	Hippocampus	−28	−32	−4	5.83	
R	STG (BA 22/21)	44	−32	0	3.29	12
R	MFG (BA 46)	56	28	32	3.93	6
R	Insula	44	0	16	2.72	3
R	STG (BA 42)	68	−36	20	2.64	2

Note: L=left, R=right, FFG=fusiform gyrus, IFG=inferior frontal gyrus, op=pars opercularis, tri=pars triangularis, orb=pars orbitalis, IOG=inferior occipital gyrus, mCC=middle cingulate gyrus, MFG=middle frontal gyrus, MOG=middle occipital gyrus, MTG=middle temporal gyrus, SFG=superior frontal gyrus, SMA=supplementary motor area, SOG=superior occipital gyrus, SPL=superior parietal lobule, STG=superior temporal gyrus.

**Table 3:** Brain activation for the high control>low control contrast during the baseline scan and after TMS was applied to OP and LIFG.

**Activation peak**			**x**	**y**	**z**	***Z***	**Voxel**	
**Baseline high>low control**								
L	IFG (tri; BA 45)		−52	24	20	5.86		511
L	SMA (BA 6)		−4	16	56	4.22		90
R	IFG (orb; BA 47)		36	28	−8	4.27		54
R	Cerebellum		12	−80	−32	3.58		29
R	IFG (tri)		44	24	24	3.49		21
R	SMA		12	12	48	2.99		5
L	MTG (BA 21)		−52	−40	0	3.02		4
R	Cerebellum		20	−80	−48	2.72		2

**OP high>low control**								
L	IFG (orb)		−48	44	−8	4.80		355
L	SMA (BA 6)		−4	20	60	4.15		76
R	Cerebellum		20	−80	−32	3.36		29
L	MTG (BA 21)		−52	−40	−4	3.59		18
R	IFG (orb)		28	32	−4	3.21		5
R	IFG (tri; BA 45)		48	24	12	2.80		2

**IFG high>low control**								
L	IFG (tri; BA 45)		−52	28	12	6.06		807
R	Cerebellum		16	−84	−32	5.44		326
R	Insula		32	24	−4	5.39		254
L	SMA (BA 6)		−4	20	56	6.10		243
L	MTG (BA 21)		−56	−48	4	3.73		96
L	Thalamus		−4	−24	−4	3.99		95
L	FFG (BA 37)		−28	−40	−20	3.93		54
L	IOG		−27	−92	−8	3.23		22
R	IOG		40	−92	−4	2.97		4
R	Midbrain		20	−24	−8	3.06		3
L	Postcentral gyrus (BA 4)		−52	−12	44	2.74		2
L	Pallidum		−12	4	−4	2.78		2

Note: L=left, R=right, FFG=fusiform gyrus, IFG=inferior frontal gyrus, op=pars opercularis, tri=pars triangularis, orb=pars orbitalis, IOG=inferior occipital gyrus, mCC=middle cingulate gyrus, MTG=middle temporal gyrus, SMA=supplementary motor area. Co-ordinates in MNI space.
