# Supplementary material for: Charting the effects of TMS with fMRI: Modulation of cortical recruitment within the distributed network supporting semantic control
Source: Neuropsychologia. 2016 Dec;93(Pt A):40–52. doi: 10.1016/j.neuropsychologia.2016.09.012 (PMC5155664; doi:10.1016/j.neuropsychologia.2016.09.012)
Supplement: Supplementary Figure 1 — Supplementary material [file mmc1.docx]

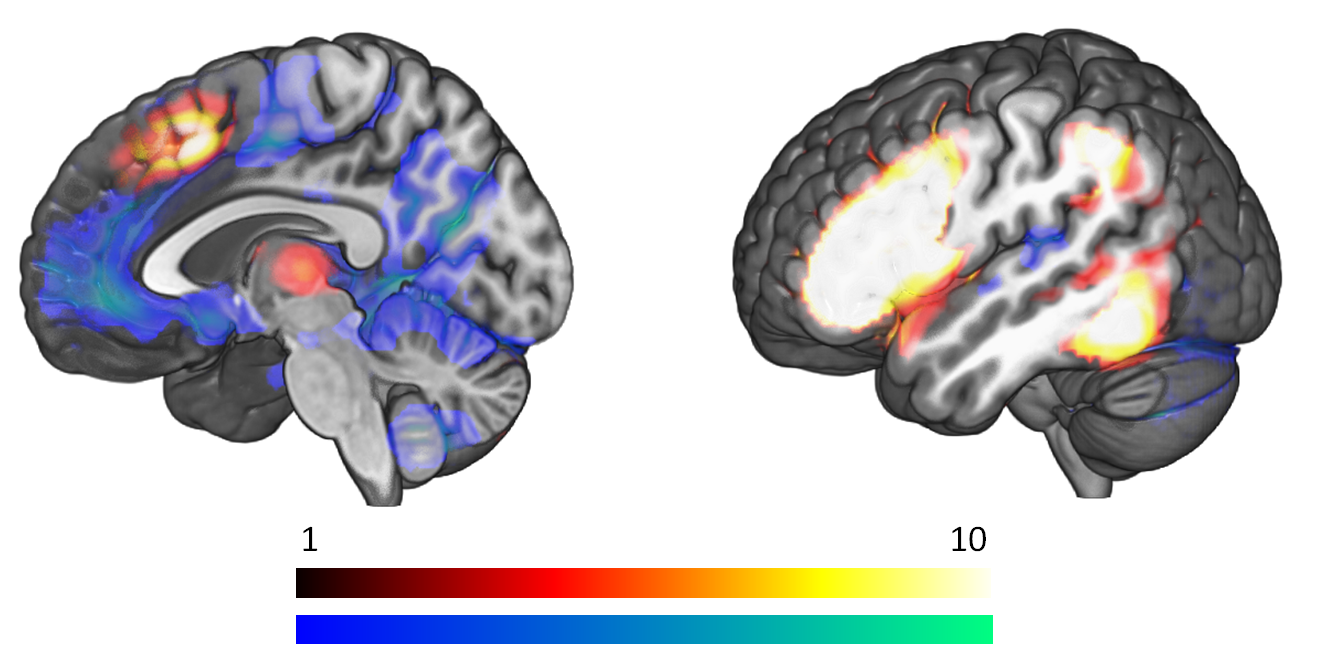


Figure S1 – Anti-correlation of the LIFG stimulation site in an independent analysis of 162 resting state datasets. The mPFC shows anti-correlation with the LIFG (blue/green) and therefore represents a region that is not functionally coupled to LIFG (positive correlation red/yellow).

Resting state datasets were collected from an independent sample of healthy young participants (age 18-35) at the University of York (these data have been partially reported elsewhere; Krieger-Redwood et al., 2016; Medea et al., 2016; Davey et al., 2016). The 9-minute resting state scan used single-shot 2D gradient-echo echo planar imaging (EPI) with a flip angle = 90°, matrix size = 64 × 64, voxel size = 3 mm3, and field of view (FOV) = 192 mm2, TR = 3000 ms, TE = 29 ms, 60 slices, 180 volumes. An intermediary FLAIR scan with the same orientation as the functional scans was collected to improve the co-registration between subject-specific structural and functional scans. Data were processed in FSL. Brain extraction from the skull was performed using the BET toolbox for both the FLAIR and the structural T1 weighted images and these scans were registered to standard space using FLIRT. Prior to conducting the functional connectivity analysis the following pre-statistics processing was applied to the resting state data; motion correction using MCFLIRT; slice-timing correction using Fourier-space time-series phase shifting; non-brain removal using BET; spatial smoothing using a Gaussian kernel of FWHM 6mm; grand-mean intensity normalisation of the entire 4D dataset by a single multiplicative factor; high pass temporal filtering (Gaussian-weighted least-squares straight line fitting, with sigma = 100 s); Gaussian low pass temporal filtering, with sigma = 2.8 s. The LIFG site for each participant formed the centre of a 3mm radius from which the time series was extracted and used as explanatory variables in a separate subject level functional connectivity analysis. In these analyses, we also included 11 nuisance regressors: the top five principal components extracted from white matter (WM) and cerebrospinal fluid (CSF) masks in accordance with the compcor method and six motion parameters. The WM and CSF masks were generated by segmenting each individual’s high-resolution structural image (using FAST in FSL). The default tissue probability maps, referred to as Prior Probability Maps (PPM), were registered to each individual’s high-resolution structural image (T1 space) and the overlap between these PPM and the corresponding CSF and WM maps was identified. Finally, these maps were thresholded (40% for the SCF and 66% for the WM), binorised and combined. The six motion parameters were calculated in the motion-correction step during pre-processing. Movement in each of the three Cartesian directions (x, y, z) and rotational movement around three axes (pitch, yaw, roll) were included for each individual. No global signal regression was performed.


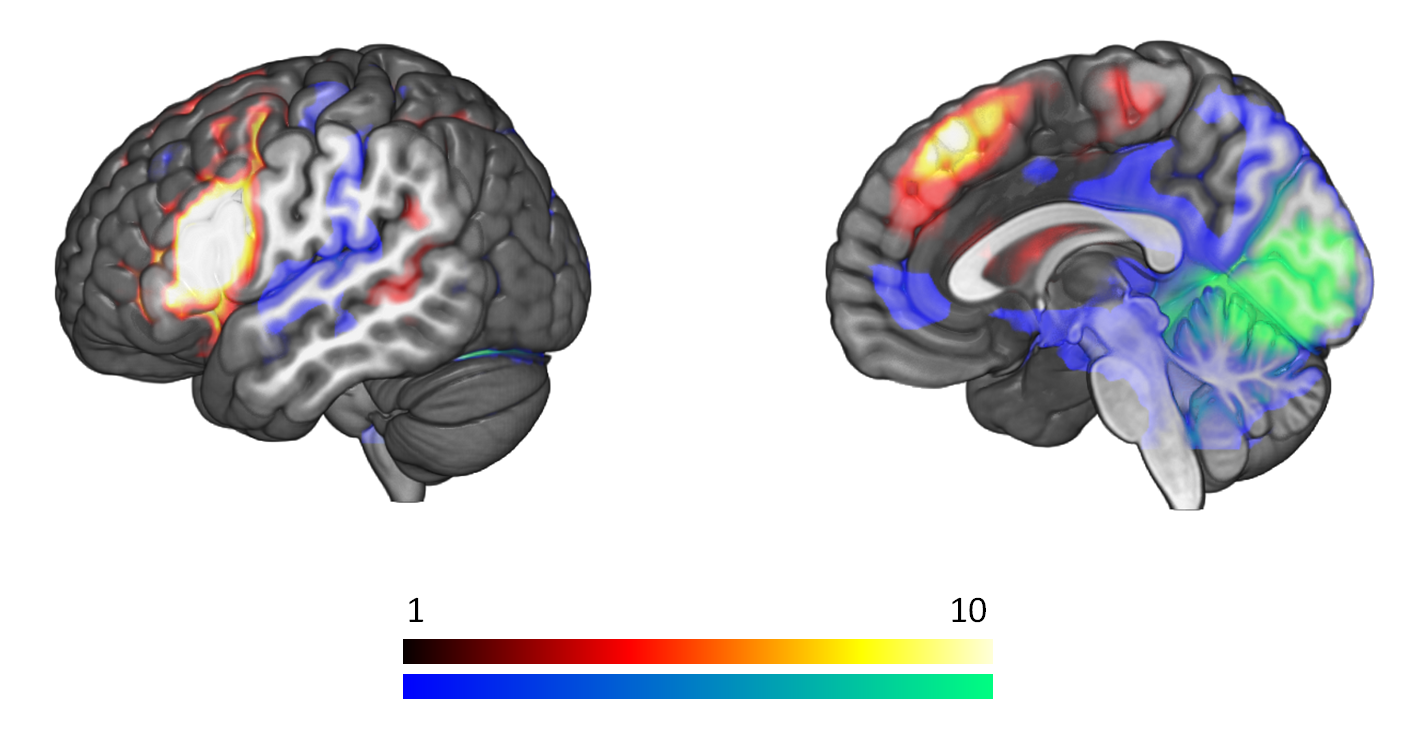


Figure S2: Psychophysical interaction examining functional connectivity of the IFG stimulation site with the OP control seed (red/yellow) and the inverse contrast (blue/green).

Note: OP is not anti-correlated with LIFG in resting-state functional connectivity analyses (see Supplementary Figure 1), unlike surrounding brain areas, and unlike the mPFC control region used in the PPI analysis presented in the main manuscript. This may explain why this supplementary analysis showed a less extensive area of coupling between LIFG and pMTG. Importantly, however, we continued to observe greater functional connectivity between LIFG and pMTG during the task, compared with the implicit baseline, in both analyses (irrespective of the choice of baseline region).
